# 2126 Optimizing a technique for visualizing retinal and choroidal blood flow noninvasively

**DOI:** 10.1017/cts.2018.104

**Published:** 2018-11-21

**Authors:** Paras Vora, Nicholas Bell, Romulo Albuquerque, Jooyoung Cho, Gregory Botzet

**Affiliations:** 1 Center for Clinical and Translational Science, University of Kentucky; 2 Department of Ophthalmology, University of Kentucky

## Abstract

OBJECTIVES/SPECIFIC AIMS: Diabetic retinopathy is an increasingly prevalent disease, difficult to screen for across the globe. We have developed and began optimizing an innovative technique to visualize and quantify retinal blood flow, to elucidate the role of the choroid in retinal pathologies such as diabetic retinopathy or choroidopathy. METHODS/STUDY POPULATION: Preliminary retinal was obtained from a surgical retina video library (Truvision, Goleta, CA, USA). Videos of different organs were recorded while vessels were occluded via a blood pressure cuff, using consumer-grade digital video cameras (NEX-5T, a7sii; Sony, New York, NY, USA). All other retinal videos were taken using a fundus camera (50×; Topcon, Oxland, NJ, USA) modified to support the above digital video cameras. All videos were processed using experimental software (MATLAB, Mathworks, Natick, MA, USA). RESULTS/ANTICIPATED RESULTS: Video imaging of the retina was optimized for lighting conditions and software requirements. Parameters were defined for the software imaging pipeline, such as frequency range of interest, sampling rate, and noise minimization. Software was developed to stabilize frames, accounting for eye saccades. Use of a biosensor enabled accurate measurement of pulse waveform, increasing signal-to-noise ratio. The optimal light requirements were determined such that adequate exposure of the retina is reproducible yet still comfortable for use in human subjects. DISCUSSION/SIGNIFICANCE OF IMPACT: This novel technique allows for an inexpensive, noninvasive, and reproducible ocular blood flow imaging platform. By optimizing this technique, we can proceed with our future plans for a pilot study to compare our imaging technique with the current standard, paving the way for future clinical studies.

